# Outcome in Patients Resuscitated following Myocardial Infarction with Acute Kidney Injury

**DOI:** 10.7150/ijms.45686

**Published:** 2020-05-29

**Authors:** Vojko Kanic, Robert Ekart, Zlatka Kanic

**Affiliations:** University Medical Center Maribor, Maribor, Slovenia.

**Keywords:** acute kidney injury, cardiac arrest, resuscitation, myocardial infarction, outcome, percutaneous coronary intervention

## Abstract

**Background:** Data on acute kidney injury (AKI) in patients with myocardial infarction (MI) who underwent percutaneous coronary intervention (PCI) after cardiac arrest are scarce. The prevalence of AKI, as classified by the Kidney Disease: Improving Global Outcomes (KDIGO) criteria; and its possible association with 30-day mortality were assessed.

**Methods:** Data on 6387 patients with MI, 342 (5.3%) with out-of-hospital cardiac arrest or arrest immediately after admission before PCI, were retrospectively analyzed. The AKI and no-AKI groups were compared. The 30-day mortality was determined.

**Results:** Ninety-three (27.2%) patients suffered AKI. AKI KDIGO stages 1, 2 and 3 occurred in 45 (13.2%), 8 (2.3%) and 40 (11.7%) patients, respectively. Higher mortality was found in AKI patients [56 (60.2%) vs. no-AKI patients 32 (12.9%); p<0.0001]. More patients died in the higher AKI KDIGO stages. In AKI KDIGO stages 1/2 and stage 3, 20 (37.7%) patients and 36 (90.0%) patients died, respectively compared to 32 (12.9%) no-AKI patients; p<0.0001.

AKI was the strongest predictor of 30-day mortality (adjusted OR 6.98; 95% CI 3.42 to 14.23; p<0.0001). Other predictors were bleeding, cardiogenic shock, contrast volume-to-glomerular filtration rate ratio, and female sex. The adjusted OR for AKI KDIGO stages 1/2 and stage 3 were 3.68; 95% CI 1.53 to 8.32; p=0.002 and 29.10; 95% CI 8.31 to 101.88; p<0.0001, respectively.

**Conclusion:** In patients resuscitated after MI undergoing PCI, AKI had a deleterious impact on the prognosis. A graded increase in the severity of AKI according to the KDIGO definition was associated with a progressively increased risk of 30-day mortality.

## Introduction

The incidence and survival of patients after out-of-hospital cardiac arrest (OHCA) vary 10-fold because of different definitions of OHCA and in-hospital cardiac arrest 1-7. The different definitions might denote diverse patient populations (patients who died suddenly, all patients who died outside of hospital, those who were resuscitated, those who were attended by medical professionals, those with witnessed arrest, traumatic cardiac arrest, etc.) 1,8, and, therefore, different survival rates would be expected. Survival rates following cardiac arrest vary from 0.6% to 25% 7. Most cases of cardiac arrest (55-78%) have a cardiac etiology. In “non-cardiac” cardiac arrests, asphyxia and respiratory causes predominate 7,9,10.

The complex combination of myocardial dysfunction and cerebral and whole-body ischemia in conjunction with the reperfusion response may lead to different combinations of multiorgan failure and significant morbidity and mortality 11. Myocardial injury and circulatory failure account for most deaths in the first three days, but ischemic brain injury is the leading cause of in-hospital death 12. The pathologic process that caused cardiac arrest in MI patients with OHCA may be resolved by coronary artery reperfusion and the survival rate in patients with percutaneous coronary intervention (PCI) is, therefore, likely to be greater than in other OHCA patients 11,13. Age, sex, time of cardiac arrest, and time to resuscitation are known predictors of survival in all OHCA patients 9. In patients with cardiac arrest due to myocardial infarction (MI), age, female sex, increased serum glucose, ST-elevation MI, and initial admission to a hospital that was competent to perform primary PCI were associated with survival 14. Data on additional risk factors and comorbidities associated with the outcome in MI patients with PCI are scarce 14.

Cardiopulmonary collapse after cardiac arrest and its aggressive treatment in MI patients are associated with increased risk of acute kidney injury (AKI) 11. Myocardial infarction in combination with post-cardiac arrest myocardial dysfunction causes hypoperfusion of the kidneys 11,15. PCI with possible atheroembolism, exposure to contrast media, bleeding, the need for cardiac support devices and drugs may further damage the kidney 15. Additionally, the systemic ischemia/reperfusion response with inflammation and microcirculatory failure further aggravates AKI 11.

Different definitions of AKI in patients with OHCA of diverse etiology were used in previous studies. No data exist on the incidence of AKI according to the Kidney Disease Improving Global Outcomes (KDIGO) guidelines and its impact on outcomes in patients with cardiac arrest due to MI who underwent PCI 16. We aimed to evaluate the prevalence of AKI, as classified by the KDIGO criteria, and to assess the impact of AKI on the outcome in patients with MI undergoing PCI who were resuscitated before hospitalization or immediately after admission but prior to PCI.

## Methods

**Figure 1:**
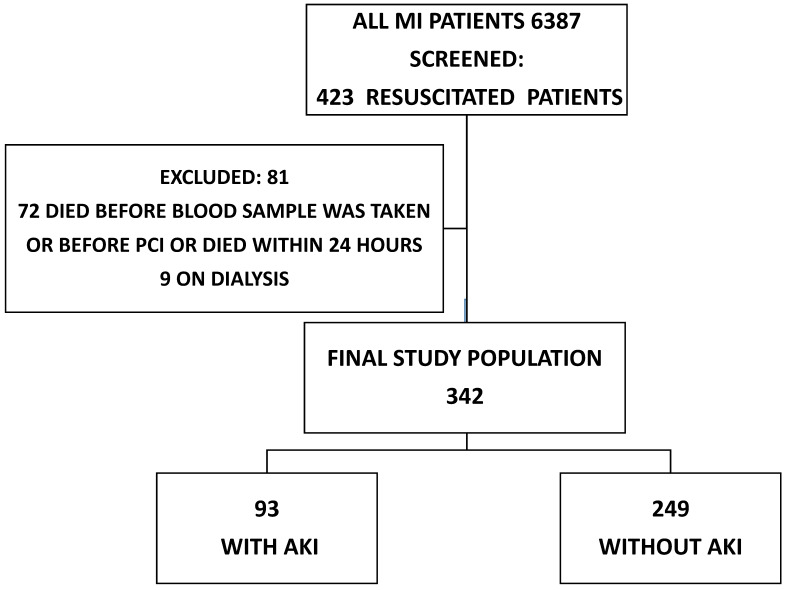
The diagram shows the number of MI patients screened, excluded and enrolled in the study. AKI: acute kidney injury; MI: myocardial infarction, PCI: percutaneous coronary intervention.

We performed a retrospective review of 6387 patients over 18 years of age with MI treated with PCI who were admitted to an academic tertiary care center between January 2007 and December 2016. We screened 423 (6.6%) patients who were resuscitated before hospitalization or immediately after admission but prior to PCI. Patients on dialysis (9 patients) and patients who died before blood samples were taken or PCI was performed [53 (12.5%) patients] were excluded. An additional 19 (4.2%) patients who died within the first 24 hours were excluded, since it was assumed that there was no time for AKI to occur, leaving 342 (81.0%) for further analysis (Figure 1).

Thrombolysis was not used. The group with AKI was compared with the group without AKI.

All-cause 30-day mortality and the incidence of AKI according to the KDIGO definition were assessed.

### Definitions

Cardiac arrest was defined as cardiopulmonary collapse with associated loss of pulses requiring chest compressions or rescue shock by a health professional. We did not exclude patients on the basis of their initial cardiac rhythm or the duration of advanced cardiac life support.

All patients were Caucasians and were treated according to the guidelines for the management of MI 17,18. The definition of ST-elevation MI was based on the ESC guidelines 17. Therapeutic hypothermia with sedation/relaxation, cold fluid infusion and external cooling with ice packs was encouraged. The temperature was maintained between 32 and 34 °C for 24 hours.

Patient baseline creatinine concentrations were defined using one of three methods. The reference serum creatinine value was defined as a concentration obtained within 3-month period before admission. For patients without this reference value, we used the minimum of either serum creatinine at the time of admission, or we used a calculated serum creatinine concentration using the MDRD equation as recommended by the Acute Dialysis Quality Initiative (ADQI) in patients without chronic kidney disease 2,19,20.

Renal dysfunction was defined as an estimated glomerular filtration rate (GFR) less than 60 ml/kg/1.73m^2^. The Modification of Diet in Renal Disease Study formula was used for calculations of GFR 21. Serum creatinine levels were determined on admission and after 24 and 48 hours. After the first 48 hours, serum creatinine levels were obtained at various time points, at the discretion of the treating physician.

AKI was defined as an increase in serum creatinine after PCI of ≥ 0.3mg/dl (26.5 μmol/L) in the first 48 hours 22. Additionally, we determined AKI using the KDIGO criteria and staged for severity 16. The Mehran score was calculated as proposed previously 23.

For calculations of AKI and mortality, we combined AKI KDIGO stages 1 and 2 (AKI KDIGO stage1/2) since there were only eight patients in AKI KDIGO stage 2 and there was no difference in 30-day mortality between AKI KDIGO stages 1 and 2 (p=0.98).

The nonionic contrast agent iopamidol (concentration 370 mg/ml) was used in our laboratory.

Renal protection with continuous infusion of saline (1ml/kg/h during PCI and for the next 12 hours) was used. In patients with overt heart failure, the hydration rate was reduced at the discretion of the operator and/or attending physician.

Heart failure was defined according to the clinical criteria (bilateral pulmonary rales, S_3_ gallop, jugular venous distension) and/or ejection fraction <30 % and/or interstitial or alveolar edema requiring diuretic therapy on chest X-ray. The ventricular ejection fraction was assessed by bedside echocardiography in the first 48 hours after admission.

Cardiogenic shock on admission was defined according to clinical and hemodynamic criteria, including hypotension (systolic blood pressure ≤90 mm Hg for ≥30 minutes or the need for supportive measures to maintain a systolic blood pressure of > 90 mm Hg) and evidence of end-organ hypoperfusion.

The Bleeding Academic Research Consortium (BARC) bleeding criteria and BARC 3a bleeding (Hb drop of 30-50 g/L or any transfusion) were used 24. All medical records were obtained from the hospital information system to complete the data collection. The study was approved by the local medical ethics committee. Data on dates of death were provided by the Slovenian National Cause of Death Registry.

### Outcome

The end point was 30-day all-cause mortality. The secondary end-point was the incidence of AKI.

### Statistical methods

Distributions of continuous variables in the two groups were compared using either the 2-sample t-test or the Mann-Whitney test. Distributions of categorical variables were compared using the chi-square test. All p-values were two-sided; values less than 0.05 were considered statistically significant. We counted the end-point events that occurred during the follow-up period and compared their rates in the cohorts of patients with and without AKI. We used binary logistic regression to identify independent predictors of AKI and to calculate the adjusted odds of 30-day mortality. Binary logistic regression models were performed using the Enter mode. The models were adjusted for age, gender, diabetes, hypertension, bleeding, intra-aortic balloon pump, mechanical ventilation, cardiogenic shock, the contrast volume-to-estimated glomerular filtration rate ratio (contrast volume/GFR), renal dysfunction on admission, and AKI. Only mechanical ventilation and cardiogenic shock on admission were used as covariates. Data were analyzed with the SPSS 23.0 software for Windows (IBM Corp., Armonk, NY).

## Results

### Descriptive patient data

The clinical study encompassed 342 patients who were resuscitated out of hospital or immediately after admission before PCI. Information on the contrast volume used during PCI was available for 310 (90.6%) patients. Other data were available for all patients.

Ninety-three (27.2%) patients suffered AKI. AKI stage 1 occurred in 45 (13.2%) patients, AKI stages 2 and 3 in eight (2.3%) and 40 (11.7%), respectively. Patients with AKI were older and were more likely to be mechanically ventilated and/or in cardiogenic shock at presentation. They more often suffered from diabetes and renal dysfunction but had less hyperlipidemia. They were more prone to bleeding, and a higher contrast volume/GFR ratio was observed. More intra-aortic balloon pumps were inserted. AKI patients were less liable to receive bivalirudin and double antiplatelet therapy. Higher peak troponin levels were usually seen together with lower ejection fraction. The Mehran score was more likely to be higher in the AKI group.

Therapeutic hypothermia was used in 141 (41.2%). There was no difference in the rates of AKI between the temperature treatment groups (52.7% in patients treated with therapeutic hypothermia compared to 38.6% in no-hypothermia patients; p=0.08). Therapeutic hypothermia was not associated with AKI, (adjusted OR 0.63; 95% CI 0.52 to 1.58). Additionally, therapeutic hypothermia did not affect the need for renal replacement therapy [7(3.5% patients in the hypothermia group compared to 8(5.6%) in the no-therapeutic hypothermia group; p=0.48].

Renal replacement therapy (RRT) [14 (4.1%) of all patients] was exclusively used in AKI patients. Continuous veno-venous hemofiltration was required by 10 patients, three patients were treated with a combination of veno-venous hemofiltration and temporary hemodialysis and one patient with temporary hemodialysis. Only one patient needed permanent RRT (hemodialysis). The risk of dying in 30 days was greater in patients who required RRT (71.4% of patients with RRT died in 30 days compared to 20.6% of patients without RRT). However, in patients with AKI, RRT patients had similar rates of dying compared to no-RRT patients (71.4% vs. 54.8%; p=0.38). The basic clinical and procedural characteristics of patients differed significantly between the groups, as shown in Tables 1 and 2.

### Mortality

After 30 days, 88 (25.7%) resuscitated patients had died. Patients who suffered AKI had a higher 30-day mortality [56 (60.2%)) patients with AKI died compared to 32 (12.9%) patients without AKI; p<0.0001]. AKI independently predicted a worse 30-day mortality (adjusted OR 6.98; 95% CI 3.42 to 14.23; p<0.0001). Other predictors of mortality were bleeding, cardiogenic shock, contrast volume/GFR ratio and female sex (Table 3).

AKI KDIGO Stage 1/2 patients had a higher unadjusted 30-day mortality [20 (37.7%) patients with AKI 1/2 died compared to 32 (12.9%) in the no-AKI group; p<0.0001)].

The majority of AKI KDIGO stage 3 patients died within 30 days [36 (90.0%) patients died compared to 32 (12.9%) patients without AKI: p<0.0001)]. The adjusted OR for AKI KDIGO stage 1/2 and stage 3 were 3.68; 95% CI 1.53 to 8.32; p=0.002 and 29.10; 95% CI 8.31 to 101.88; p<0.0001, respectively.

### Predictors of AKI

Bleeding (OR 2.34; 95% CI 1.30 to 4.19; p=0.004), cardiogenic shock (OR 1.90; 95% CI 1.01 to 3.58; p=0.046), mechanical ventilation (OR 2.91; 95% CI 1.59 to 5.23; p=0.001), and contrast volume/GFR ratio (OR 1.20; 95% CI 1.01 to 1.41; p=0.033) predicted AKI.

Interestingly, renal dysfunction did not independently predict AKI (adjusted OR 1.43; 95% CI 0.63 to 2.49).

## Discussion

Data on the association between AKI according to the KDIGO guidelines, and outcomes in patients with MI who underwent PCI after resuscitation are very limited 14. In our study, almost seven percent of all MI patients undergoing PCI were resuscitated out of hospital or immediately after admission before PCI. We validated the prevalence of AKI based on the KDIGO criteria in unselected resuscitated patients with MI treated with PCI and their association with the outcome. The main findings of the present investigation are as follows:

- Patients who suffered AKI after PCI had an almost seven times greater risk of dying in 30 days;
- AKI was the strongest independent predictor of mortality;
- A graded increase in the severity of AKI according to the KDIGO definition is associated with a progressively increased risk of 30-day mortality in these patients.

Our finding, that almost 30% of patients suffered AKI is consistent with a previous finding 2 but lower than the incidence of AKI in most previous observations of OHCA patients 12. Our study group was specific because all patients suffered MI and all underwent PCI, which was not the case in previous reports, where different OHCA patients with or without PCI were included in the study 12. The pathologic process that led to OHCA in our patients could have been resolved by PCI. Post-cardiac arrest myocardial dysfunction is both responsive to therapy and reversible, which could explain the better survival rate and the lower incidence of AKI 11.

We found AKI to be the strongest predictor of 30-day mortality in multivariate analysis, thus confirming the findings of a previous study in a similar population 15,25. Yanta et al. found higher mortality in resuscitated patients with AKI, but AKI did not predict the outcome 2. Unfortunately, any comparison is difficult due to the different study populations, different definitions of AKI and different treatment. Comparisons must be made in light of these differences. In our opinion, these differences could explain our result, which is in line with the previous finding 25.

Even patients with AKI KDIGO stage 1/2 were almost four times more likely to die within 30-days compared to patients without AKI, while 90% of patients with AKI KDIGO stage 3 died within 30 days. A graded increase in the severity of AKI according to the KDIGO definition was associated with a progressively increased mortality risk. Our study confirmed the previous findings that bleeding, cardiogenic shock, and mechanical ventilation predict AKI 15,26,27.

The contrast volume/GFR ratio was independently associated with AKI as found previously 28-30. The contrast volume/GFR ratio probably better discriminates between the patients who are at higher risk of AKI than the contrast volume used or GFR alone 29,30. The contrast volume used was similar in both groups and was not associated with AKI, while the contrast volume/GFR ratio differed significantly between the groups and predicted both AKI and 30-day mortality. Besides contrast volume and GFR, AKI in such patients after resuscitation is a consequence of the interplay of hemodynamic and nephrotoxic pathways, inflammation, and whole-body ischemia, in conjunction with the reperfusion response 11,26.

Renal dysfunction on admission did not predict AKI. GFR on admission in the AKI group (median 61.0 ml/min/1.73m2) was above the defined threshold for renal dysfunction. This is consistent with the previous observation that renal dysfunction does not predict AKI in MI patients with cardiogenic shock and/or mechanical ventilation 15,31. It is of note that 40% of patients with renal dysfunction in our analysis presented with cardiogenic shock, more than half of them were mechanically ventilated, and they were more liable to bleed after PCI (46%), all of which predisposed to AKI 15,17,18,27,32,33.

There was a trend towards a higher rate of AKI in patients with therapeutic hypothermia (p=0.08). It is noteworthy that patients treated with therapeutic hypothermia more often suffered STEMI (91.8% vs. 83.1%; p=0.021), presented more often mechanically ventilated (69.2% vs. 21.9%; p<0.0001) and in cardiogenic shock (33.6% vs. 16.4%); p<0.0001), and bled more (56.2% vs. 20.2%; p<0.0001), which are all known risk factors for AKI 30,34. Neither AKI nor RRT were associated with therapeutic hypothermia, as found previously 35,36.

Female sex did not predict AKI but predicted a worse outcome, as seen previously 14.

Mechanical ventilation on admission predicted AKI but was not associated with mortality in our analysis. Patients with mechanical ventilation on admission presented more often in cardiogenic shock (32.7% vs. 18.8%, p=0.004), bled more (52.0% vs. 22.4% p<0.0001), and had a greater contrast volume/GFR ratio (2.94 vs. 2.38; p=0.021), which makes the higher mortality in these patients understandable. Although mechanical ventilation is a lifesaving procedure for critically ill patients, it is, at the same time, an indicator of a worse clinical outcome 37. In resuscitated patients, mechanical ventilation is often only a temporary measure to overcome cardio-circulatory collapse and not a necessity because the patients are critically ill. These patients can often be weaned from mechanical ventilation shortly after the cause of cardiac arrest has been resolved. Only mechanical ventilation on admission was included in the multivariate analysis in the study. This difference may explain our finding.

Our findings have some potential clinical implications. We can easily identify factors that signify highly vulnerable patients on admission after OHCA. These are female sex, cardiogenic shock, and mechanical ventilation. Early commencement of preventive strategies like radial access to prevent bleeding, measures to ensure optimal hemodynamics, crystalloid infusions, and low contrast volume should be encouraged. Furthermore, adjustment of antithrombotic drugs and discontinuation of nephrotoxic drugs may ensure a better outcome. Additionally, consultation with a nephrologist early in the course of treatment might be beneficial 15,16,26,28,30. In contrast to most studies, there was no difference in treatment between the groups with regard to reperfusion therapy. It is therefore questionable whether we can extrapolate our results to all OHCA patients.

In conclusion, we found that resuscitated patients with MI after PCI who suffered an AKI have a higher risk of dying. Preventive measures should be implemented early in the treatment.

### Limitations

This study has several limitations. This was a relatively small, retrospective, single-center study. The data on the “time to resuscitation”, “time to return of spontaneous circulation” and the “time between cardiac arrest and PCI” were not available for a sufficient number of patients to be considered in the evaluation, variables with known impact on AKI. We did not follow the data on possible normalization of serum creatinine. The exact hydration rate was not available in all patients because, in patients with overt heart failure, the hydration rate was reduced at the discretion of the operator and/or attending physician. Data on medications (ACE inhibitors, ARB, spironolactone), hyperuricemia and glucose were not collected. Moreover, data on a history of heart failure, coronary heart disease, and atrial fibrillation were not collected. The data on contrast volume were missing for some patients. However, patients without data on contrast volume were similarly distributed between the groups. Therefore, it is highly unlikely that this would have a significant impact on our result. We did not collect data for the Killip class or blood pressure, which are variables with a strong impact on AKI. Furthermore, we lack the data on urine output for 7 days. Calculations of eGFR were based on creatinine on presentation, which may not have been in a steady state, and thus may not be a true estimate of patients' baseline kidney function. For the calculations of eGFR, we used the MDRD equation, whose accuracy and reliability decrease with extreme eGFR values. Moreover, we were able to follow only all-cause mortality. Only the Caucasians with MI who underwent PCI, were included in the analysis. Therefore, the generalizability of our result is questionable.

## Conclusion

AKI after PCI has a deleterious impact on the prognosis in patients resuscitated following MI. A graded increase in the severity of AKI according to the KDIGO definition is associated with a progressive increase in the risk of 30-day mortality.

## Figures and Tables

**Table 1:** Basic patient characteristics

	AKI; N = 93	No AKI; N = 249	p
Age, years∞	66.0 (12.5)	61.8 (11.8)	0.029
Male gender, N (%)*	67 (72.0)	177 (71.1)	0.89
ST-elevation MI, N (%)*	83 (89.2)	215 (86.3)	0.59
Diabetes, N (%)*	25 (26.9)	35 (14.1)	0.01
Hypertension, N (%)*	45 (48.4)	109 (43.8)	0.46
Hyperlipidemia, N (%)*	18 (19.4)	86 (34.5)	0.008
Renal dysfunction, N (%)*	46 (49.5)	53 (25.3)	<0.0001
Cardiogenic shock, N (%)*	37 (39.8)	48 (19.3)	<0.0001
Mechanical ventilation, N (%)*	63 (67.7)	87 (34.9)	<0.0001
Creatinine, mgl/dL^¥^	1.20 (0.94, 1.65)	0.94 (0.79, 1.20)	<0.0001
GFR, ml/kg/1.73m^2 ¥^	61.0 (40.6, 79.2)	76.6 (61.2, 96.1)	<0.0001
Mehran score, N∞	12.9 (5.4)	8.66 (5.2)	<0.0001
BMI, N∞	27.8 (4.5)	27.4 (4.5)	0.49

∞ Mean (standard deviation); * Comparison made using the chi-square test; ¥ Median (25th, 75th percentile). BMI: body mass index; GFR: glomerular filtration rate; MI: myocardial infarction; N: number.

**Table 2:** Procedural characteristics of patients

	AKI (N = 93)	No AKI (N = 249)	p
Hypothermia, N (%)*	49 (52.7)	96 (38.6)	0.08
Contrast volume, ml^¥^	152.5 (114.7, 197.5)	150.0 (112.0, 200.0)	0.89
Contrast volume-to-GFR ratio^¥^	2.66 (1.67, 4.19)	1.90 (1.34, 2.90)	0.001
Bivalirudin, N (%)*	6 (6.5)	40 (16.1)	0.02
GPIIb/IIIa, N (%)*	58 (52.4)	133 (53.4)	0.14
Dual antiplatelet therapy, N (%)*	63 (67.7)	221 (88.8)	<0.0001
PCI LMCA, N (%)*	8 (8.6)	13 (5.2)	0.31
PCI LAD, N (%)*	45 (46.4)	115 (46.2)	0.72
PCI LCX, N (%)*	26 (28.0 )	57 (22.9)	0.33
PCI RCA, N (%)*	21 (22.6)	72 (28.9)	0.28
Radial access, N (%)*	5 (5.4)	23 (9.2)	0.37
IABP, N (%)*	11 (11.8)	13 (5.2)	0.054
TIMI flow 0/1 after PCI, N (%)*	17 (6.8)	11 (11.8)	0.18
BARC 3a bleeding, N (%)*	52 (55.9)	69 (27.7)	<0.0001
Heart failure, N (%)*	76 (81.7)	123 (53.4)	<0.0001
Peak troponin, (mol/L)^ ¥^	40.92 (8.3, 84.3)	24.26 (6.7, 65.6)	<0.0001
Ejection fraction, (%)∞	33.2 (7.3)	39.2 (9.9)	<0.0001
Renal replacement therapy, N (%)*	14 (15.1)	0 (0)	<0.0001

∞ Mean (standard deviation);* Comparison made using the chi-square test; ¥ Median (25th, 75th percentile). AKI: acute kidney injury; BARC: Bleeding Academic Research Consortium; GFR: glomerular filtration rate; GPIIb/IIIa: GPIIb/IIIa receptor inhibitor; IABP: intra-aortic balloon pump; LAD: left anterior descending artery; LCX: circumflex artery; LMCA: left main coronary artery; N: number; PCI: percutaneous coronary intervention; RCA: right coronary artery; TIMI 0/1: TIMI grade flow after PCI: 0/1.

**Table 3:** Independent predictors of 30-day mortality

Variable	OR	95% CI	p
AKI	6.98	3.42 to 14.23	<0.0001
Bleeding	2.47	1.18 to 5.17	<0.0001
Cardiogenic shock	2.82	1.38 to 5.75	0.004
Female sex	2.46	1.21 to 5.01	0.013
Contrast volume-to-GFR ratio	1.33	1.08 to 1.63	0.007

AKI: acute kidney injury; GFR: glomerular filtration rate.

## Abbreviations

AKI: acute kidney injury
BARC: Bleeding Academic Research Consortium
BMI: body mass index
CI: confidence interval
ESC: European Society of Cardiology
GFR: glomerular filtration rate
GPIIb/IIIa: GPIIb/IIIa receptor inhibitor
IABP: intra-aortic balloon pump
KDIGO: Kidney Disease Improving Global Outcomes
LAD: left anterior descending artery
LCX: circumflex artery
LMCA: left main coronary artery
MI: myocardial infarction
N: number
OHCA: out of hospital cardiac arrest
OR: odd ratio
PCI: percutaneous coronary intervention
RCA: right coronary artery
RRT: renal replacement therapy
TIMI: Thrombolysis in myocardial infarction
